# Roles of clinical supervisors in the clinical training of radiography students in Zambia: a qualitative study

**DOI:** 10.4314/ahs.v22i2.73

**Published:** 2022-06

**Authors:** Osward Bwanga, Edward Mwansa

**Affiliations:** 1 Midland Regional Hospital at Tullamore, Radiology Department, Co. Offaly, Ireland. o.bwanga@yahoo.com; 2 Evelyn Hone College of Applied Arts and Commerce, School of Applied and Health Sciences, Lusaka, Zambia. eddiemwansa3@gmail.com

**Keywords:** clinical supervisor, clinical training, radiographer, radiography student, Zambia

## Abstract

**Introduction:**

Clinical supervisors are responsible for the facilitation of practice-based learning for radiography students. However, literature is scarce on the roles of clinical supervisors in the clinical training of students in Zambia.

**Objective:**

This study was aimed at identifying and exploring the roles of clinical supervisors in the clinical training of radiography students in Zambia.

**Methods:**

A qualitative design with unstructured interviews was used in this study. Ten individual interviews were conducted in July 2018 with clinical supervisors of radiography students working at the main clinical training and placement sites of the Lusaka and Copperbelt provinces of Zambia, respectively. All digital interview recordings were transcribed verbatim and analysed thematically.

**Results:**

Three main roles of a clinical supervisor emerged: managerial, educational, and supportive. The managerial role deals with organising and managing clinical training resources. The educational role involves imparting knowledge and skills to students. The supportive role involves supporting students with social and learning problems. Findings show the inter-relationship of these roles to each other.

**Conclusion:**

Clinical supervisors need to understand their roles in order to develop and maintain their competences in the facilitation of practice-based learning. This could also help stakeholders to provide appropriate support to clinical supervisors.

## Introduction

Clinical supervision is an essential component of health and social care education programmes. It allows students to translate theory into practice under the clinical supervision of experienced practitioners. 1–4 In radiography, designated radiographers who facilitate the learning process of students are often called clinical supervisors. The Health Workforce Australia 2 define a clinical supervisor as a practitioner who is responsible for the day-to-day supervision of a student during clinical training. Previous studies in radiography have reported the role of a clinical supervisor as complex due to the competing demand for providing patient care whilst simultaneously facilitating the learning processes of students. 5–7 According to the College of Radiographers, 8 the roles of clinical supervisors include developing teaching and supervisory skills, offering a level of supervision appropriate to the competence and experience of a student, and providing learning opportunities to radiography students.

Radiography literature identifies direct and indirect clinical supervision as the two main approaches employed in the facilitation of practice-based learning for students.^9^,^10^ Until a student has achieved the required competence in each examination, all assignments are carried out under the direct supervision of a clinical supervisor. 7,10 During indirect supervision, a clinical supervisor must be immediately available to assist the student regardless of their level of competence. 10,11 Indirect supervision is mostly reserved for final year radiography students as they are presumed to have reached a sufficient level of competence. 7,11

There are currently three Higher Education Institutions (HEI) offering undergraduate radiography programmes in Zambia: Evelyn Hone College (EHC), Lusaka Apex Medical University (LAMU), and University of Zambia (UNZA). EHC is a technical institution and offers a three-year diploma in radiography which was established in 1970. LAMU is a private medical university and offers a four-year Bachelor of Science degree in radiography which started in 2011. In 2018, UNZA also established a five-year Bachelor of Science degree in radiography due to a shortage of radiographers and an increase in demand for imaging services. 12 All three training programmes are accredited by the Health Professions Council of Zambia (HPCZ).

In Zambian radiography education system, first-year students are wholly involved in preclinical courses, whilst the rest undertake theory on campus and clinical training at the University Teaching Hospital (UTH) of Lusaka and other hospitals. Students begin their clinical training in their second year which runs in a block system. Clinical supervisors in each affiliated radiology department provide practice-based learning for students. The eligibility criteria for a supervisor include: being a radiographer with more than two years clinical working experience and holding a professional qualification equal to or higher than that of the students under supervision.^13^–^15^

Literature is scarce on this subject in Zambia. To the knowledge of the researchers, this is the first study to investigate the roles of clinical supervisors in the facilitation of practice-based learning for radiography students in Zambia. The increasing knowledge and understanding in this area could help clinical supervisors to identify areas in which they need to develop and maintain their competencies. This study was, therefore, aimed at identifying and exploring the roles of clinical supervisors in the clinical training of radiography students in Zambia.

## Methods

### Study design and settings

A qualitative exploratory research design was used to identify and explore the roles of clinical supervisors of radiography students. This study was part of a larger study focusing on clinical supervision strategies to support radiographers. This first phase of the research project was conducted at the main radiography clinical training sites of the Lusaka and Copperbelt provinces of Zambia. These study sites were selected because they are the main teaching hospitals, which are affiliated with the schools of radiography and offer a range of imaging services to support students' learning.

### Study population and sampling

The population (N=60) was radiographers who are working and facilitating the learning processes of students at the two study sites. The researchers, being educators and experts in this field, purposively selected the participants until saturation point (N=10). Newell and Burnard^16^ define purposive sampling as a non-probability sampling in which participants are chosen according to the researcher's judgement as to their suitability for this study. The inclusion criteria was supervisors working at the study sites with more than two years clinical working experience. However, clinical supervisors working outside the two study sites were excluded.

### Data collection procedures

Data were collected in July 2018 by means of unstructured face-to-face interviews focusing on the role clinical supervisors play in the clinical training of radiography students. This involved the researcher questioning participants to discover their understanding of the topic under investigation. 16,17 The main question each participant was asked was “What are your roles in the clinical training of radiography students?”. This was followed by probing questions. The main question was given to all participants in advance of their interviews to enable them to reflect on their experiences. This strategy helped in having detailed answers. The saturation point was reached after conducting and interviewing 10 participants. Polit and Beck 18 define data saturation as the point where no new information emerges from the interviews. Table 1 shows the demographic characteristics of the participants.

**Table 1:** Demographic characteristics of participants (N=10)

Characteristic	Value	Frequency
Work location	Copperbelt province	4
	Lusaka province	6
Position or rank	Radiographer	5
	Senior radiographer	1
	Principal radiographer	2
	Chief radiographer	2
Work experience in the clinical training of radiography students	2–10 years	2
	11–15 years	4
	> 16 years	4

All interviews were audio-recorded with each participant's permission and supplemented with field notes. The average duration of the interviews was 30 minutes per participant.

### Data management and analysis

Audio-recordings were transcribed verbatim by the lead researcher and the co-author to begin the process of data analysis. This allowed them to get “closer” to the data. 16 The Braun and Clarke 19 five phase thematic framework was used to analyse data manually (Table 2).

**Table 2:** Data analysis using Braun and Clarke thematic framework

Stage	Activity
Stage 1: Familiarisation with data	The process involved actively repeating the reading of the data by searching for meanings and patterns iteratively and making notes on printed copies of the transcripts
Stage 2: Generating initial codes	This was conducted manually and aimed at identifying a particular feature of the data set
Stage 3: Searching for themes	The aim was to reduce the number of codes by searching for common elements to raise them to the level of higher-order codes, commonly known as themes
Stage 4: Reviewing themes	This involved reviewing and refining the themes in line with the objective of this study
Stage 5: Defining and naming themes	This stage involved defining and further refining the themes. “Define and refine” refers to identifying the essence of what each theme was about and determining what aspect of the data each theme captures 19

### Establishment of rigour

The Lincoln and Guba model of trustworthiness as described by Polit and Beck, 18 was used to evaluate the rigour for this study. This model was implemented by adhering to four concepts of trustworthiness: credibility, transferability, dependability, and confirmability. To ensure credibility, the lead researcher familiarised himself with the study sites before data collection.^17^,^18^ Data was triangulated through collection from two different hospitals and the co-author performed a peer-debriefing to counter check the themes generated. 16 After the transcription was completed, the lead researcher went back to participants, to ascertain whether the transcribed data was an accurate version of their words.^18^ Trasferability was achieved through a dense description of the research process. 18,20 To ensure dependability, the lead researcher adopted an “auditing” approach by keeping complete records of all phases of the research process. 20 Lastly, to enhance confirmability, direct quotes from participants have been included to support the study findings. 18 Additionally, a detailed description has been included to enable the reader to establish how the findings accurately portray participants' responses.

### Ethical considerations

Permission to conduct this study was sought and obtained from the Tropical Diseases Research Centre (TDRC) research ethics committee (reference no: TDRC/C4/02/2018). The details of the study were explained, and informed consent obtained from each participant. To maintain anonymity, each participant was assigned a number, and this was used instead of their names. Confidentiality was ensured by keeping all documents under lock and key. Furthermore, data stored in digital format were also secured with encryption and passwords. Each participant was informed of the right to withdraw from participation without penalty.^18^

## Results

Three main roles of a clinical supervisor of radiography students emerged in this study: managerial, educational, and supportive (Table 3).

**Table 3:** Roles of a radiographer as clinical supervisor of radiography students

Theme	Category	Sub-category
**Theme** **1**: Managerial role of a clinical supervisor of radiography students	-Manager	-Communication with schools of radiography
		-Identification of radiographers to supervise radiography students
		-Orientation of students to the radiology department
		-Organisation and management of training resources
		-Supervision of radiographers and students
Theme 2: Educational role of a clinical supervisor of radiography students	-Clinical teacher	-Impart knowledge and skills to students
		-Guide students on how to apply theory into clinical practice
	-Role model	-Serve as a role model for radiography students
	-Feedback provider	-Provide feedback to radiography students
		-Provide feedback to the schools of radiography
	-Clinical assessor	-Assess radiography students on their clinical performance
**Theme** **3**: Supportive role of a clinical supervisor of radiography students	-Student supporter	-Support students with social problems
		-Support students with learning difficulties

### Theme 1: Managerial role of a clinical supervisor of radiography students

Participants stated that they were responsible for organising and managing training resources in conjunction with their hospital management. These were consumables needed during clinical training such as X-ray films, processing chemicals, contrast media agents, ultrasound gel, and cleaning materials:

“*My role is to ensure that students have the necessary equipment, utensils and accessories to use during clinical training*” (Participant 7).

Welcoming and orientating new students to the radiology department was reported by participants as part of the managerial role of a clinical supervisor:

“*My role is to orientate new radiography students. The orientation process occurs by showing the students the equipment we have and their design. I also show them where to register the patients and where the resultant X-ray images are evaluated before they are dispatched*” (Participant 10).

Three roles of a chief radiographer (manager) were also identified: communicating with the schools of radiography, identification and assignment of suitable radiographers to supervise students, and overall supervision of these radiographers:

“*I am like a focal person who links the radiology department with the schools. My role is overall supervision. That is, identifying the radiographers who are under my charge who can supervise students*” (Participant 3).

The chief radiographers also ensure that the imaging equipment and accessories are in good working condition.

### Theme 2: Educational roles of a clinical supervisor of radiography students

Four educational roles of a clinical supervisor were identified: clinical teacher, role model, feedback provider, and assessor.

### Educational role 1: clinical teacher

During the interviews, participants identified themselves as clinical teachers of radiography students. They reported imparting knowledge and skills to students and guiding them on how to apply theory into practice:

“*My role is to mentor students in imaging equipment operation, radiographic techniques, patient care, professionalism, and radiation protection*” (Participant 8).

The teaching of patient care, specifically infection control, to radiography students was identified as part of the clinical teaching role of a clinical supervisor:

“*My speciality is in infection prevention, so I teach and ensure that students adhere to all patient infection prevention measures*” (Participant 2).

Participants also reported teaching professional behaviours to radiography students:

“*Verbally I teach them what is and is not accepted in this profession using my experience. I also base my teaching on code of professional conduct for radiographers issued by the Radiological Society of Zambia (RSZ)*” (Participant 6).

The teaching of professionalism to students was incorporated into daily activities.

### Educational role 2: role model

Participants identified themselves as role models for radiography students:

“*I lead by example for them to follow*” (Participant 5).

One participant identified role modelling as one of the roles of a clinical supervisor. Sadly, the participant claimed to be a poor role model:

“*Sometimes I lack commitment, I feel too lazy to attend to the students, which doesn't make me a role model*” (Participant 4).

Participants stated that they are examples that students emulate in their behaviour.

### Educational role 3: feedback provider

During the interviews, participants reported responsibility for providing feedback to radiography students on their clinical performance. The feedback provided was based on direct observation of students' performances:

“*I provide feedback to students immediately if I observe that the student has not behaved in a professional manner*” (Participant 2).

Feedback was also sent to the schools of radiography at the end of students' clinical attachments in the form of a report which was prepared by the chief radiographer:

“*We also give feedback to the school on the challenges we face, which may cause the students to perform not as expected so that as they go back, the school will know what the institution is lacking and maybe from there they can pick it up and know how to help the students. At the end of the attachment, it is important to give feedback because it helps to fill in the gaps, the schools will know whether there is a need to improve the curriculum*” (Participant 3).

### Educational role 4: clinical assessor

Assessment of students on their clinical competence was reported by participants as one of the roles of a clinical supervisor:

“*I give them simple exams at the beginning of their clinical training to assess their knowledge before placement*” (Participant 9).

“*My role is assessing students. This is usually done through observation of daily work activities, sometimes without the awareness of students during clinical practice. But there is also a time when we prepare some exams for the students at the end of their attachments*” (Participant 4).

For assessments, participants reported using direct observations of students' clinical performances and practical record books.

### Theme 3: supportive role of a clinical supervisor of radiography students

Participants identified themselves as supporters of radiography students with social problems and learning difficulties. They reported supporting students with social problems, such as accommodation:

“*I remember the time I had to support a student who came for attachment and didn't know that accommodation was not being provided by the institution. He had nowhere to go, so I took him home and stayed with him until he got settled and found accommodation for himself*” (Participant 5).

Participants also reported supporting students with learning difficulties and sickness:

“*But for those who present learning difficulties, I provide flexibility in the delivery of the lessons*” (Participant 1).

“*There are also certain students who fall sick, so it is my responsibility to see that a student is seen by the medical doctor without paying*” (Participant 7).

The support resulted in developing a good working relationship between clinical supervisors and radiography students. However, participants reported a lack of guidelines for supporting students with learning difficulties and disabilities.

## Discussion

Three main roles of clinical supervisors of radiography students were identified: managerial, educational, and supportive. This finding agrees with the literature.^2^,^10^,^11^,^21^ These roles are interrelated and multifaceted, whereby clinical supervisors transform themselves to fit the needs of their students. The term “chameleon” has been used in the literature to describe how clinical supervisors switch from one role to another during the same clinical teaching encounter. 22 Our study also revealed that clinical supervisors changed their roles according to the situation.

### Managerial role of a clinical supervisor of radiography students

Creating a conducive learning environment is the real substance of the clinical training of radiography students.^10^,^11^ Clinical departments should have adequate facilities and enough clinical supervisors to supervise students.^5^,^8^ The College of Radiographers 8 also states that clinical placement providers have a responsibility to ensure that new students have a full and formal induction. Walsh 3 explains that the orientation reduces anxiety in students and increases motivational learning through the early identification of learning opportunities. Our study found that new students to the clinical department were met by the chief radiographer on the first day of their placement. The meeting started the orientation process by introducing students to staff, showing them the available imaging equipment and departmental workflow.

This study found that clinical supervisors were responsible for organising and managing imaging equipment and accessories. A recent study conducted by Thompson and Taylor 7 also found supervising radiographers to be managers of clinical training resources. This role is vital in the provision of quality clinical training for radiography students and radiology services.^8^ Proper management is required because imaging equipment and supplies, as well as clinical teaching and learning resources, are expensive. Good management will also value the preservation and availability of imaging equipment when needed.^23^ The misuse of equipment or supplies wastes money and increases healthcare costs. Ehrlich and Coakes^24^ state that a radiographer who organises and manages imaging resources adequately and avoids wastage demonstrates high standards of professional behaviour.

### Educational roles of a clinical supervisor of radiography students

In our study, the first educational role of a clinical supervisor identified was that of a clinical teacher. Harden and Laidlaw 25 define clinical teaching as teaching which focuses on real patients in the clinical setting. Students are taught radiographic techniques, equipment operation, patient care, radiation protection, and professionalism using an integrated approach during routine imaging and care of patients. The role of imparting knowledge and skills to radiography students and guiding them on how to apply theory into practice has been reported in the radiography literature.^5^,^7^ Walsh 3 points out that clinical teaching is best delivered when there are an understanding and application of the educational principles relating to the failitation of practice-based learning.

The role model was the second educational role of a clinical supervisor identified in our study. In a previous study by Conway et al., 26 radiography students also identified supervising radiographers as their role models. Within the clinical learning environment, the value of role models as an additional influence in the development of students' professional identities is significant. 26,27 Role models inspire and teach by example, often whilst doing other activities. 3,26,27 This means that students learn by being with and watching their clinical supervisors' attitudes and skills. Everything a clinical supervisor does, whether positive or negative, is likely to be regarded by students as acceptable professional behaviour or practice.^6^,^27^

Providing feedback to radiography students was the third educational role of a clinical supervisor identified in this study. A study conducted by Kayembe and Bwanga^28^ also found providing feedback to radiography students to be one of the roles of supervising radiographers. Our study found that feedback was provided to students based on direct observation, took place immediately after the observation of a behaviour, and was given privately. Feedback enables students to recognise their deficiencies, highlights what is expected, and has a motivating effect.^3^,^25^,^28^ Providing constructive feedback to students is one of the competencies that clinical supervisors may not have covered during their pre-service training. This is one area where clinical supervisors may need to develop their competencies.

The fourth educational role of a clinical supervisor identified in our study was that of an assessor or examiner. Clinical supervisors have a responsibility to protect the profession and members of the public by assessing students' competence.^29^ Assessment of clinical performance is a teaching activity that requires clinical supervisors to apply educational principles and competencies which are not on the radiography curriculum in Zambia. This is another area where clinical supervisors of radiography students may need to develop and maintain their competencies.

### Supportive role of a clinical supervisor of radiography students

This study revealed that clinical supervisors supported students with social problems and learning difficulties. The role of supporting students was also identified in a review conducted by Chamunyonga and others. 30 This role also involves supporting students being bullied, harassed, or stressed. 3,21 In our study, the most common social problem reported was accommodation. According to Maslow's hierarchy of needs, learning is a higher-order need of self-actualisation, while physiological or biological needs are lower-order needs. 3 This means that clinical supervisors should support lower-order needs, such as accommodation, as these must be resolved before the student can concentrate on training. Slow learners and sickness were also common learning problems identified where clinical supervisors supported radiography students. However, a lack of written guidelines regarding supporting students with learning difficulties and disabilities was reported as a challenge in supporting students. Murphy 31 suggests the need to have guidelines and a trained individual responsible for supporting students with learning difficulties.

## Conclusion

This study has identified three roles of clinical supervisors of radiography students: managerial, educational, and supportive. Understanding these roles could help clinical supervisors to identify areas where they need to develop and maintain the competencies of clinical supervision. The current study investigates the clinical supervisor roles from the perspective of the supervisors. Future studies need to investigate clinical supervisor roles from the perspective of radiography students.
